# Autophagy-related long noncoding RNAs can predict prognosis in patients with bladder cancer

**DOI:** 10.18632/aging.103947

**Published:** 2020-11-07

**Authors:** Cong Lai, Zhenyu Wu, Juanyi Shi, Kaiwen Li, Jiamin Zhu, Zhenhong Chen, Cheng Liu, Kewei Xu

**Affiliations:** 1 Department of Urology, Sun Yat-Sen Memorial Hospital, Sun Yat-Sen University, Guangzhou, China

**Keywords:** bladder cancer, autophagy, lncRNA, risk score, prognosis

## Abstract

We investigated whether autophagy-related long noncoding RNAs (lncRNAs) can predict prognosis in bladder cancer. We obtained bladder cancer lncRNA data from The Cancer Genome Atlas and autophagy-related genes from the Human Autophagy Database. Fifteen autophagy-related lncRNAs with prognostic significance were identified. Multivariate Cox analysis was used to construct a risk score model, which divided bladder cancer patients into high-risk and low-risk groups. We found that patients in the low-risk group had better survival than those in the high-risk group. Subgroup analysis showed that patients in the high-risk group also had worse OS than that in the low-risk group in subgroups based on age, gender, clinical stage, and TNM stage. We next established a nomogram according to the results of multivariate Cox regression, which included age, gender, clinical stage, TNM stage, and risk score. The area under the curve for 3- and 5-year overall survival predicted by the nomogram were 0.711 and 0.719, respectively. Bioinformatics analysis demonstrated that the 15 identified lncRNAs are involved in the cell cycle, DNA replication, cell adhesion, cancer pathway, WNT signaling pathway, and oxidative stress. These findings confirm that autophagy-related lncRNAs are predictive of prognosis in bladder cancer patients and may affect tumor progression.

## INTRODUCTION

Bladder cancer is the tenth most common malignant tumor and the second most common urological malignancy of the world with approximately 549,000 new cases and 200,000 deaths in 2018 [1]. Seventy percent of patients diagnosed with bladder cancer have non-muscle-invasive bladder cancer, whereas the rest have muscle-invasive bladder cancer (MIBC) [2]. MIBC has a high recurrence rate and poor prognosis, with a 5-year survival rate of < 50% [3]. In addition, most bladder cancer patients are diagnosed with mid- or late-stage disease due to the lack of symptoms or signs of disease. MIBC is difficult to treat, and patients often experience recurrence, causing substantial physical, mental, and financial burdens [4]. Accurate prognostic and prediction tools for bladder cancer exist [5]. However, few studies have validated the predictive performance of these prognostic models, and novel biomarkers are needed to improve the utility of prediction tools for bladder cancer.

Autophagy contributes to tumor cell homeostasis by degrading and recycling damaged or unnecessary cytoplasmic components [6]. However, autophagy overactivation may promote the autophagic death of tumor cells, similar to apoptosis [7]. Long noncoding RNAs (lncRNAs) are a group of RNAs that participate in human physiological and pathological processes by interacting with other biological molecules. During autophagy, lncRNAs act as a molecular sponge and adsorb miRNA, avoiding the inhibitory effect of miRNA on mRNA translation and regulating the expression of autophagy-related genes (ATGs) [8]. Moreover, lncRNAs can directly target the promoter region of ATG [9] or recruit other molecules to promote gene transcription [10], thereby regulating cell autophagy. Recently, abundant abnormally expressed lncRNAs were found to be a biomarker for early diagnosis and prognosis of bladder cancer [11].

Therefore, autophagy-related lncRNAs may have value as prognostic biomarkers in bladder cancer. Wu et al. showed that the lncRNA UCA1 decreased miR-582-5p expression and promoted bladder cancer progression and drug resistance through ATG7-mediated autophagy inhibition [12]. In addition, autophagy-related lncRNAs contribute to the pathogenesis of bladder cancer and could serve as diagnostic molecular markers [13].

The value of lncRNAs as prognostic indicators has been validated in several cancers [14–16]. However, there have been no studies of autophagy-related lncRNAs as prognostic indicators in bladder cancer. In this study, we established a prognostic model for bladder cancer based on autophagy-related lncRNAs and explored the biological functions of autophagy-related lncRNAs in cancer.

## RESULTS

### Acquisition of autophagy-related lncRNAs

**Figure 1:**
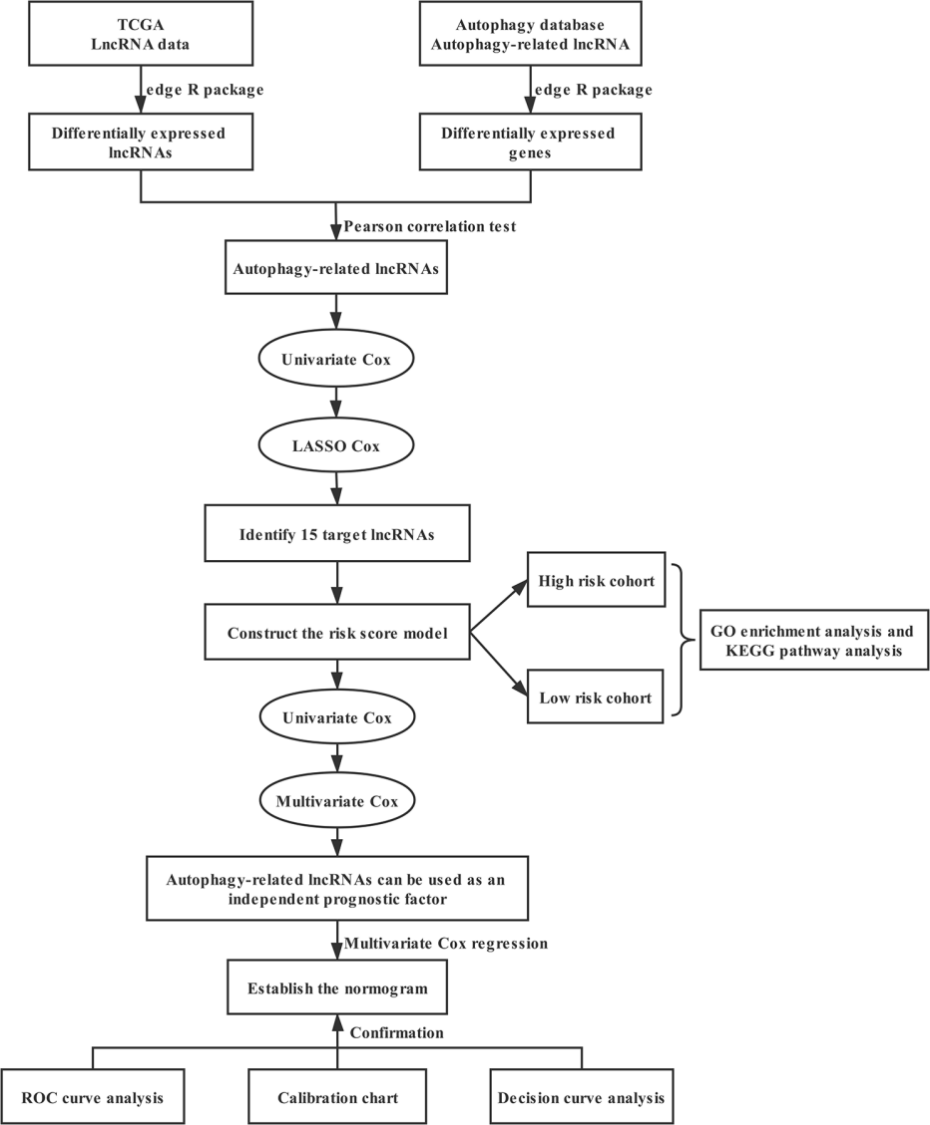
**Flowchart showing the creation and evaluation of the prognostic model.**

**Figure 2:**
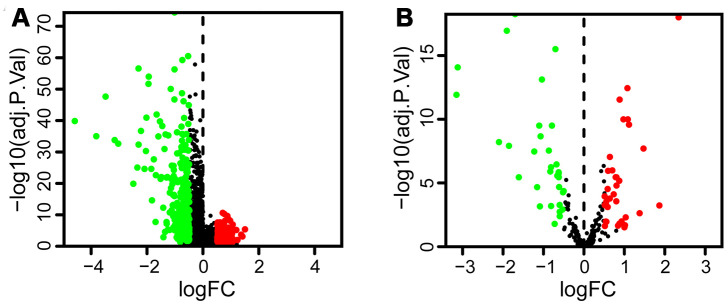
**The volcano plot shows the differential expression of lncRNAs and autophagy-related genes in bladder tumor compared to paracancerous tissues.** Red dots and green dots represent significantly upregulated and downregulated lncRNAs and autophagy genes, respectively, whereas black dots indicate no difference. (**A**) The volcano plot demonstrates that 161 lncRNAs were upregulated and 293 were downregulated in bladder tumor. (**B**) The volcano plot shows that 34 autophagy-related genes were upregulated and 32 were downregulated in bladder tumor.

This study was designed to investigate the prognostic significance of autophagy-related lncRNAs in bladder cancer, as shown in the flowchart in Figure 1. LncRNA sequencing data and corresponding clinical data of bladder cancer, including 411 tumors and 19 paracancerous tissues, were obtained from The Cancer Genome Atlas (TCGA) database. Two hundred thirty-one autophagy-related genes were extracted from the Human Autophagy Database (HADb) (Supplementary Table 1). We set | log_2_FC | > 0.5 and false discovery rate (FDR) < 0.05 as the thresholds to recognize the differentially expressed genes and lncRNAs in tumors compared to paracancerous tissues. We found 454 differentially expressed lncRNAs, of which 161 were upregulated and 293 were downregulated in tumors (Figure 2A). We also identified 66 differentially expressed autophagy-related genes, 34 of which were upregulated and 32 downregulated in tumors (Figure 2B). Seventy-seven autophagy-related lncRNAs were determined using Pearson correlation analysis (Table 1).

**Table 1:** Correlation between the prognostic autophagy genes and lncRNAs in bladder cancer.

**Autophagy gene**	**LncRNA**	**Correlation**	***p* value**	**Regulation**
BIRC5	AC099850.3	0.518139105	1.30E-29	positive
CAPN10	AGBL5-IT1	0.424576114	2.04E-19	positive
FOXO1	MIR29B2CHG	0.435964391	1.70E-20	positive
CD46	MIR29B2CHG	0.407547076	7.06E-18	positive
CLN3	AL355353.1	0.425152699	1.80E-19	positive
MAPK8IP1	AC136475.2	0.46171729	4.30E-23	positive
CAPN10	AC024075.2	0.473146691	2.56E-24	positive
HSPA5	AC024075.2	-0.419571858	5.90E-19	negative
MAPK8IP1	DNM3OS	0.412718741	2.46E-18	positive
HSPB8	AC005180.2	0.472544439	2.98E-24	positive
CAPN10	SNHG12	0.440382796	6.31E-21	positive
RAB24	SNHG12	0.404207973	1.38E-17	positive
CAPN10	AL023284.4	0.429340224	7.29E-20	positive
HSPA5	AL023284.4	-0.430036211	6.27E-20	negative
FOXO1	PWAR6	0.540110317	1.69E-32	positive
ITPR1	FENDRR	0.405810179	1.00E-17	positive
PTK6	AL133355.1	0.43976867	7.25E-21	positive
CAPN10	AL117379.1	0.428480048	8.79E-20	positive
MAPK8IP1	MAGI2-AS3	0.462169543	3.86E-23	positive
CAPN10	AC092171.4	0.52453338	1.98E-30	positive
HSPB8	AC005180.1	0.466961353	1.20E-23	positive
CAPN10	AL139089.1	0.51464032	3.59E-29	positive
NFKB1	SNHG10	-0.404200697	1.38E-17	negative
CDKN2A	LINC00294	0.442008415	4.37E-21	positive
EEF2K	AC024075.1	0.478725257	6.23E-25	positive
PTK6	AC021016.2	0.405175822	1.14E-17	positive
ITGB4	BLACAT1	0.436552419	1.49E-20	positive
MAPK8IP1	AP001107.5	0.50671687	3.42E-28	positive
CAPN10	AC016773.1	0.42249368	3.18E-19	positive
MAPK8IP1	AC104794.2	0.439202182	8.23E-21	positive
MAPK8IP1	AC084033.3	0.519598763	8.49E-30	positive
CAPN10	AL391244.3	0.475046212	1.59E-24	positive
CAPN10	PTOV1-AS2	0.442560388	3.85E-21	positive
CAPN10	AC010542.5	0.487587387	6.23E-26	positive
MAPK8IP1	NIFK-AS1	0.491202104	2.39E-26	positive
CAPN10	AC116914.2	0.427819307	1.01E-19	positive
BAG1	B4GALT1-AS1	0.48783034	5.84E-26	positive
FOXO1	AC018521.6	0.509230871	1.68E-28	positive
SIRT1	AC018521.6	0.404261771	1.37E-17	positive
ITPR1	AC018521.6	0.447335409	1.29E-21	positive
SPHK1	MIR4435-2HG	0.562525632	1.14E-35	positive
SIRT1	PAXIP1-AS2	0.413319455	2.17E-18	positive
SPHK1	LINC02081	0.453139766	3.34E-22	positive
SPHK1	AL441992.1	0.433057059	3.23E-20	positive
CDKN2A	AL441992.1	0.430590752	5.55E-20	positive
MAPK8IP1	AL691432.2	0.478949474	5.88E-25	positive
MAPK8IP1	AC139768.1	0.441061307	5.41E-21	positive
CAPN10	AC010326.3	0.473158295	2.56E-24	positive
CD46	AC008764.2	0.443055192	3.44E-21	positive
TP73	TMPO-AS1	0.413141337	2.25E-18	positive
PTK6	AP001453.3	0.440938236	5.57E-21	positive
MAPK8IP1	AC008537.2	0.591028131	4.54E-40	positive
CCL2	PCAT19	0.461014532	5.10E-23	positive
CAPN10	MHENCR	0.432362894	3.77E-20	positive
FOXO1	SNHG14	0.507629996	2.64E-28	positive
SIRT1	SNHG14	0.410648755	3.76E-18	positive
BAK1	U62317.1	0.500377717	1.99E-27	positive
MAPK8IP1	AL662844.4	0.495330386	7.88E-27	positive
HSPB8	MBNL1-AS1	0.425183231	1.79E-19	positive
FOXO1	AC108449.2	0.495103087	8.38E-27	positive
SIRT1	AC108449.2	0.439043082	8.53E-21	positive
ITPR1	AC108449.2	0.437375631	1.24E-20	positive
FOXO3	AC108449.2	0.456092847	1.66E-22	positive
DLC1	AL136084.3	0.448830494	9.13E-22	positive
FOXO1	AC124312.5	0.571300478	5.59E-37	positive
CXCR4	LINC00926	0.743387272	1.93E-73	positive
CAPN10	AC005726.3	0.405778811	1.01E-17	positive
MAPK8IP1	FGF14-AS2	0.55493846	1.43E-34	positive
HSPB8	AC053503.4	0.433473297	2.95E-20	positive
BAK1	U62317.2	0.473424008	2.39E-24	positive
FOS	AC020916.1	0.586803704	2.17E-39	positive
CAPN10	AC110285.6	0.406367341	8.96E-18	positive
CD46	AL928654.2	0.427039797	1.20E-19	positive
FOS	AC025259.3	0.522766658	3.34E-30	positive
PTK6	AC105219.1	0.404972627	1.19E-17	positive
SIRT1	LINC00641	0.495343732	7.85E-27	positive
SIRT1	AL158212.3	0.476558722	1.08E-24	positive
EEF2	EPB41L4A-AS1	0.433208969	3.13E-20	positive
PTK6	AC018904.1	0.431407256	4.64E-20	positive
PTK6	KRT7-AS	0.401922795	2.18E-17	positive
FOXO1	AC011472.4	0.477859441	7.77E-25	positive
CAPN10	AL390719.2	0.616448755	2.22E-44	positive
CCL2	MIR100HG	0.428857255	8.10E-20	positive
DIRAS3	BX322562.1	0.426255606	1.42E-19	positive
MAPK8IP1	MEG3	0.432712864	3.49E-20	positive
SIRT1	AC024075.3	0.407642867	6.93E-18	positive
CAPN10	AL353622.1	0.443012149	3.48E-21	positive
MAPK8IP1	TRIM52-AS1	0.446745687	1.48E-21	positive

### Construction of the risk score model

**Figure 3:**
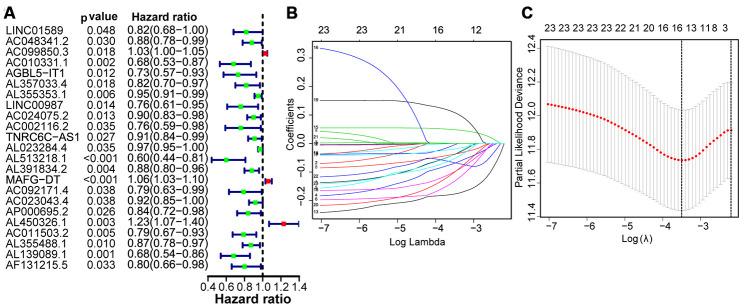
**Identification of autophagy-related lncRNAs with prognostic value.** (**A**) Risk ratio forest plot shows that 23 autophagy-related lncRNAs were significantly related to OS. (**B**) Adjusted parameters of LASSO regression model. (**C**) Illustration for LASSO coefficient spectrum of prognostic lncRNAs.

**Figure 4:**
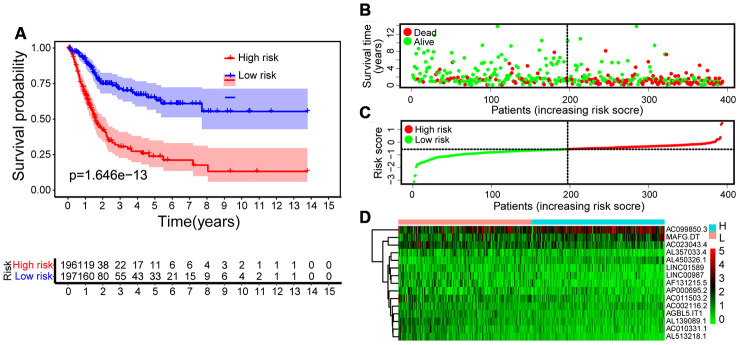
**Construction of risk score model.** (**A**) Kaplan-Meier survival analysis of bladder cancer patients shows that the high-risk group had significantly worse OS than the low-risk group. (**B**) Survival rate and survival status of bladder cancer patients. (**C**) The distribution of 15-lncRNA risk scores for each patient. (**D**) Heatmap of 15 lncRNAs in the low-risk group and the high-risk group. Cold colors represent low expression and warm colors represent high expression.

**Figure 5:**
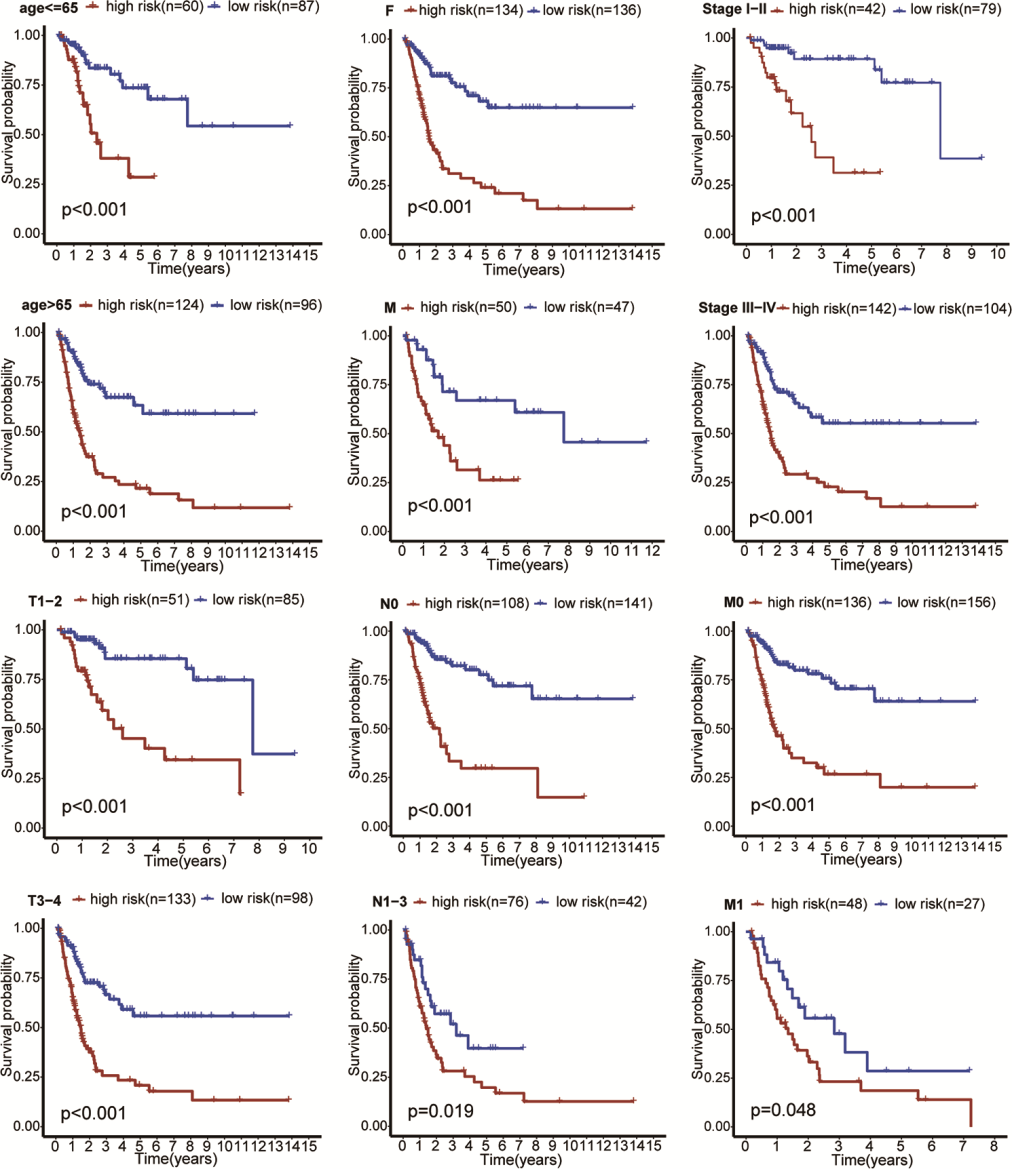
**Subgroup analysis showed that patients in the high-risk group had worse OS than that in the low-risk group in subgroups based on age, gender, clinical stage, and TNM stage.**

Using autophagy-related lncRNA and clinical data, we performed univariate Cox regression and identified 23 prognosis-associated lncRNAs (Figure 3A). We used least absolute shrinkage and selection operator (LASSO) regression analysis to further screen 15 pivotal lncRNAs (Figure 3B and 3C). Next, we used multivariate Cox regression to calculate their respective coefficients (βi) to establish a risk score model. We set the median risk score as the cutoff value and divided 411 patients into high-risk and low-risk groups. The overall survival (OS) in the low-risk group was significantly better than that in the high-risk group (P <0.001, Figure 4A). Subgroup analysis showed that patients in the high-risk group had worse OS than that in the low-risk group in subgroups based on age, gender, clinical stage, and TNM stage (Figure 5). Furthermore, our results indicated that three lncRNAs (AC099850.3, MAFG-DT, and AL450326.1) were adverse prognostic factors for bladder cancer, whereas the other lncRNAs (LINC01589, AC010331.1, AGBL5-IT1, AL357033.4, LINC00987, AC002116.2, AL513218.1, AC023043.4, AP000695.2, AC011503.2, AL139089.1, and AF131215.5) were favorable prognostic factors.

### Establishment and evaluation of the prognostic model

**Figure 6:**
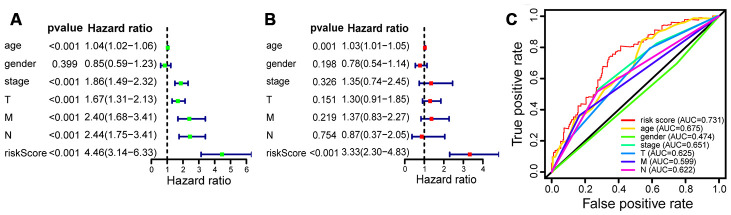
**The prognostic value of clinicopathological characteristics and risk score.** (**A**) Univariate Cox regression shows that the clinical factors of age, gender, clinical stage, TNM stage, and risk score were closely associated with OS. (**B**) Multivariate Cox analysis demonstrates that the 15-lncRNA signature is an independent prognostic factor for bladder cancer. (**C**) ROC curve analysis indicates that the 15-lncRNA signature is an excellent predictive indicator (AUC = 0.731).

The results of univariate and multivariate Cox analysis indicated that the 15-lncRNA signature was a reliable predictor of OS in bladder cancer patients. Univariate Cox regression demonstrated that the clinical characteristics of age, gender, clinical stage, TMN stage, and risk score were associated with OS (Figure 6A). We conducted a multivariate Cox analysis of these clinical characteristics and found that the 15-lncRNA signature was an independent prognostic factor for bladder cancer (P <0.001; Figure 6B). Receiver operating characteristic (ROC) curve analysis also proved that the 15-lncRNA signature was an excellent predictive indicator of prognosis (area under the curve [AUC] = 0.731; Figure 6C).

**Figure 7:**
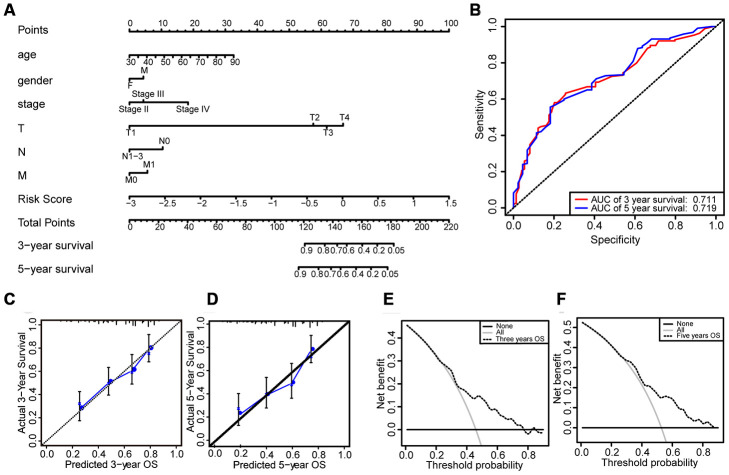
**Construction and evaluation of the prognostic model.** (**A**) Nomogram for predicting the 3- and 5-year survival rates of bladder cancer patients. (**B**) The ROC curve analysis demonstrates that the AUCs for 3- and 5-year OS predicted by the nomogram are 0.711 and 0.719, respectively. (**C**) Calibration curve based on 3-year OS of the nomogram. (**D**) Calibration curve based on 5-year OS of the nomogram. (**E**) DCA based on 3-year OS of the nomogram. (**F**) DCA based on 5-year OS of the nomogram.

Based on the results of multivariate Cox regression, we established a nomogram that included age, gender, clinical stage, TMN stage, and risk score (Figure 7A). AUCs for 3- and 5-year OS predicted by the nomogram were 0.711 and 0.719, respectively (Figure 7B), which confirmed the accuracy of the prediction. The calibration curve and decision curve analysis (DCA) of the prognostic model showed that the model had a good predictive ability (Figure 7C–7F).

### Gene set enrichment analysis

**Figure 8:**
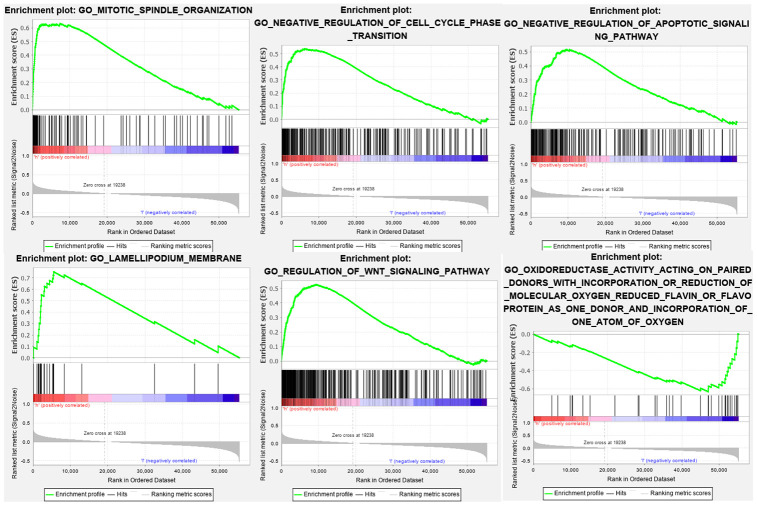
**GO enrichment analysis shows that the genes are enriched in cell division, negative regulation of cell cycle, negative regulation of apoptosis, cell migration, oxidative stress, and WNT pathway.**

**Figure 9:**
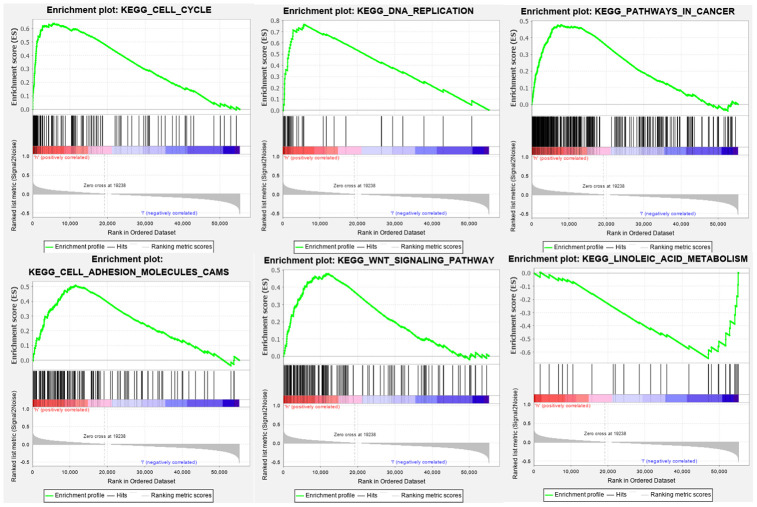
**KEGG pathway analysis indicates that these genes are involved in the cell cycle, DNA replication, cell adhesion, cancer pathway, linoleic acid metabolism, and WNT signaling pathway.**

We performed Gene Ontology (GO) enrichment analysis and Kyoto Encyclopedia of Genes and Genomes (KEGG) pathway analysis on the differentially expressed genes between the high-risk and low-risk groups. GO enrichment analysis showed that the genes were enriched in cell division, negative regulation of cell cycle, negative regulation of apoptosis, cell migration, oxidative stress, and the WNT pathway (Figure 8). KEGG pathway analysis showed that these genes were involved in the cell cycle, DNA replication, cell adhesion, the cancer pathway, linoleic acid metabolism, and the WNT signaling pathway (Figure 9). This information may help researchers to conduct future studies on the mechanisms of autophagy-related lncRNAs that affect bladder cancer pathogenesis.

## DISCUSSION

Bladder cancer is the second most common malignant tumor of the urinary system [1]. Approximately 30% of bladder cancer patients progress to MIBC, which is characterized by high rates of metastasis and recurrence and a 5-year OS < 50% [17]. Therefore, predicting bladder cancer prognosis is important to correctly stratify and treat bladder cancer patients. In this study, we developed a prognostic model based on autophagy-related lncRNAs, which had excellent prediction performance in bladder cancer.

In tumors, autophagy can maintain cell homeostasis and survival by removing nonessential and dysfunctional substances; however, autophagy can also eradicate tumor cells through activation of the apoptosis pathway [18]. lncRNAs may affect autophagy in a variety of ways [19], and studies have shown that autophagy-related lncRNAs are associated with tumor prognosis. Huang et al. showed that the lncRNA PVT1 triggered cytoprotective autophagy and promoted tumor development in pancreatic ductal adenocarcinoma and that high PVT1 expression predicted poor prognosis of patients [20]. Luan et al. identified 10 autophagy-related lncRNAs in glioma and confirmed that these lncRNAs have prognostic value in glioma patients [15]. However, autophagy-related lncRNAs in bladder cancer had not been previously studied.

In this study, we identified a 15-lncRNA signature that predicted prognosis in bladder cancer patients and constructed a prognostic model based on the signature. We found that patients in the low-risk group according to the 15-lncRNA signature had longer OS than patients in the high-risk group. According to the results of multivariate Cox regression, a nomogram was developed based on age, gender, clinical stage, TNM stage, and risk score. The AUCs for 3- and 5-year OS predicted by the nomogram were 0.711 and 0.719, respectively. The nomogram prediction model was evaluated using DCA and the calibration curve, and the results confirmed the model’s prediction efficiency.

Among the 15 vital lncRNAs, PAC099850.3, MAFG-DT, and AL450326.1 were negatively associated with OS in bladder cancer patients, whereas LINC01589, AC010331.1, AGBL5-IT1, AL357033.4, LINC00987, AC002116.2, AL513218.1, AC023043.4, AP000695.2, AC011503.2, AL139089.1, and AF131215.5 were positively associated with OC. LncRNAs have received much attention as potential prognostic markers for tumors. Zhou et al. found that high expression of AC099850.3 predicted worse survival outcomes in patients with squamous cell carcinoma of the tongue [21]. In addition, in pancreatic ductal adenocarcinoma cells, LINC01589 expression is significantly increased, and LINC01589 expression is closely associated with OS in patients with this malignancy [22]. The latest research from our center has discovered that, in bladder cancer, the exosomal lncRNA LNMAT2 stimulates the formation and migration of lymphatic endothelial cells in vitro and intensifies cancer lymphangiogenesis and lymphatic metastasis in vivo [23]. Our laboratory has planned in vivo and in vitro experiments to further investigate the functions of these lncRNAs.

To determine the functions of the lncRNAs in the 15-lncRNA signature in bladder cancer, we performed GO enrichment and KEGG pathway analyses on the genes differentially expressed in the high- and low-risk groups. Gene set enrichment analysis showed that these lncRNAs were involved in the cell cycle, DNA replication, cell adhesion, cancer pathway, linoleic acid metabolism, WNT signaling pathway, and oxidative stress. Cell proliferation and cancer pathways have long been known to participate in autophagy regulation and tumor pathogenesis [24, 25]. Furthermore, inhibition of the WNT signaling pathway in glioblastoma was reported to induce autophagic flux and consequently promote apoptosis of tumor cells [26]. Our findings demonstrate that autophagy-associated lncRNAs in bladder cancer might regulate tumor growth and progression through modulation of the cell cycle, DNA replication, cell adhesion, cancer pathway, and WNT signaling pathway.

Our study has some limitations. First, it is a retrospective study using data from the TCGA database, which lacks information on smoking history and treatment. Thus, the prognostic model was constructed using limited clinical data, restricting its predictive performance. Second, we included 15 lncRNAs in the prognostic algorithm, making it difficult to apply in clinical setting. However, the development of high-throughput sequencing may make it more feasible for a prognostic model based on multiple indicators to be applied in the clinic.

In summary, we developed a 15-lncRNA signature that can predict prognosis in patients with bladder cancer. Bioinformatics analysis suggested that autophagy-related lncRNAs may regulate tumor pathogenesis through modulation of the cell cycle, DNA replication, cell adhesion, cancer pathway, and WNT signaling pathway. Our results indicate that autophagy-related lncRNAs can predict the prognosis of bladder cancer patients and play a key role in bladder cancer biology.

## MATERIALS AND METHODS

### Sample sources and processing

We obtained information on lncRNAs and corresponding clinical data of patients with bladder cancer from the TCGA (https://cancergenome.nih.gov/). Autophagy-related genes were extracted from HADb (http://www.autophagy.lu/). We set | log_2_FC | > 0.5 and FDR < 0.05 as thresholds to recognize the differentially expressed genes and lncRNAs based on the edgeR package. We performed Pearson correlation analysis to identify autophagy-related lncRNAs with criteria of coefficients | R^2^ | > 0.5 and P < 0.05. Univariate Cox regression was performed on autophagy-related lncRNAs and clinical data to identify prognosis-related lncRNAs. We used LASSO regression analysis to identify lncRNAs closely associated with OS.

### Construction of the risk score model

LncRNAs selected by LASSO analysis were included in the multivariate Cox regression model to calculate their βi. Then, a risk score model consisting of βi and lncRNA expression levels (Expi) was established as follows: Risk score = $\sum_{i\;=\;1}^{15}(\beta_{i}*Exp_{i})$ The risk score for each patient was calculated according to the equation. In addition, we divided patients into high-risk and low-risk groups based on the median risk score. The Kaplan-Meier survival curve showed a prognostic difference between high-risk and low-risk patients. We conducted a subgroup analysis to further validate the model.

### Establishment and evaluation of the prognostic model

The risk score and clinical characteristics such as age, gender, clinical stage, and TNM stage were used in the prognostic model. A nomogram was established based on the results of multivariate Cox regression to predict each patient’s 3- and 5-year OS. We used calibration plots generated by the rms package to evaluate the properties of the nomogram. We further assessed the accuracy of the nomogram by performing ROC curve analysis to obtain AUCs. Then, the calibration curve and DCA were conducted to evaluate the model [27].

### Gene set enrichment analysis

We performed GO enrichment and KEGG pathway analyses on the genes differentially expressed between the high-risk and low-risk groups. The functions were derived by analyzing the gene set between two biological states. In addition, we explored whether the differentially expressed genes were enriched between the two groups during autophagy.

### Statistical analysis

All statistical analysis was conducted using R version 3.6.2 (Institute for Statistics and Mathematics, Vienna, Austria; https://www.r-project.org) (Package: limma, pheatmap, survival, glmnet, survminer, survivalROC, rms, foreign, timeROC). The correlation was assessed using Pearson correlation analysis. The log-rank test compared the survival curves created using the Kaplan-Meier method. The categorical variables were compared using the χ^2^ test. Univariate and multivariate Cox regression were used to analyze the correlation between clinicopathological features and risk scores and the OS of patients. ROC curves and AUCs were generated to assess the predictive power of the constructed model. The calibration curve and DCA were conducted to assess the model. Two-tailed P <0.05 was considered statistically significant.

## Supplementary Material

Supplementary Table 1
