# Supplementary material for: Autophagy-related long noncoding RNAs can predict prognosis in patients with bladder cancer
Source: Aging (Albany NY). 2020 Nov 7;12(21):21582–96. doi: 10.18632/aging.103947 (PMC7695412; doi:10.18632/aging.103947)
Supplement: Supplementary Table 1 [file aging-12-103947-s001..pdf]

SUPPLEMENTARY TABLE

Supplementary Table 1. Differentially expressed genes between tumors and paracancerous tissues.

Gene	logFC	*t* value	*p* value
ITPR1	-1.711211591	-9.99821276	5.82E-19
BIRC5	2.330867032	9.843078149	1.03E-18
TP53INP2	-1.917507989	-9.489545614	1.18E-17
PRKN	-0.715248379	-9.028062519	3.24E-16
FOS	-3.131625271	-8.562301729	8.74E-15
BCL2	-1.049274661	-8.237304029	7.86E-14
P4HB	1.065285181	7.997376566	3.74E-13
HSPB8	-3.161640094	-7.806600824	1.25E-12
GAPDH	0.872217802	7.664155888	2.96E-12
BAX	0.966250635	7.103777855	1.05E-10
RGS19	1.074943366	7.098956856	1.05E-10
BID	1.104399899	6.936292429	2.74E-10
DLC1	-1.115557486	-6.892502129	3.26E-10
SIRT1	-0.801197451	-6.884206661	3.26E-10
GABARAPL1	-1.082117387	-6.562624851	2.25E-09
CCL2	-2.110885631	-6.378216224	6.41E-09
CX3CL1	-1.863771279	-6.253419879	1.26E-08
EIF4EBP1	1.462683793	6.160490835	2.05E-08
NFE2L2	-0.878405044	-6.086864426	2.97E-08
PPP1R15A	-1.238860122	-6.049164758	3.51E-08
FKBP1A	0.631525371	5.867114109	9.35E-08
NRG2	-0.688494108	-5.61303327	3.60E-07
FOXO1	-0.823404832	-5.502139746	5.96E-07
HSPA5	0.692031605	5.384284944	1.06E-06
HGS	0.586627946	5.361258889	1.15E-06
BAG3	-0.842208961	-5.324076357	1.30E-06
EEF2K	-0.635843459	-5.290228865	1.49E-06
PEX14	-0.634120633	-5.241922521	1.84E-06
DIRAS3	-0.669057008	-5.15288417	2.80E-06
FOXO3	-0.632339382	-5.092963028	3.64E-06
FADD	0.774549309	5.088226043	3.64E-06
IKBKE	0.794189215	5.075275645	3.68E-06
MYC	-1.620344213	-5.074108273	3.68E-06
ERO1A	0.863593335	4.93224432	6.97E-06
BCL2L1	0.795356613	4.741242128	1.65E-05
EEF2	-0.624108868	-4.678351177	2.15E-05
CDKN1A	-1.1675604	-4.667755536	2.20E-05
MTMR14	0.576672037	4.591231109	3.06E-05
ST13	-0.510815487	-4.5036497	4.44E-05
NFKB1	-0.528189417	-4.448194372	5.57E-05
BAK1	0.725853364	4.350883363	8.00E-05
CAPN10	0.502578069	4.255798895	0.000115854
CLN3	0.629011097	4.15706667	0.000172694
TP73	0.789795413	4.033609467	0.000282758
HSP90AB1	0.538297421	3.991066543	0.000329945
SQSTM1	-0.610674455	-3.836860888	0.000548212
RAB24	0.50371573	3.827992199	0.000558043
CDKN2A	1.85525917	3.804735379	0.000601089

NAMPT	-0.822228314	-3.771534499	0.000664095
CXCR4	-1.098824531	-3.748958666	0.000710944
EDEM1	0.567918074	3.719100052	0.000772956
CAPN1	0.577618874	3.70837815	0.000792754
BNIP3L	-0.540217546	-3.572455587	0.001251452
MAPK8IP1	-0.608998098	-3.451784435	0.001886409
SERPINA1	1.366839907	3.376820363	0.002432076
BAG1	-0.60803663	-3.213206241	0.00417667
ITGB4	1.022664751	3.144215358	0.005132978
ERBB2	0.920099711	2.884140581	0.010763897
CD46	0.531255912	2.875118398	0.010941547
ATG9B	0.544820043	2.861400743	0.011285774
SPHK1	0.867023828	2.732212885	0.016154964
MAP1LC3A	-0.733939631	-2.711359379	0.016814849
PTK6	1.004118835	2.623276844	0.021296293
ITGA3	0.832787674	2.591118556	0.023112799
CTSD	0.507767366	2.55879707	0.024564372
TP63	0.990984207	2.456462225	0.031102513
